# Diseases of the Chest

**Published:** 1890-03-01

**Authors:** 


### THE PRACTITIONER'S MIRROR AND RETROSPECT.

### MEDICINE.

### DISEASES OF THE CHEST.

Sir James Risdon Bennett's introductory address at the London Hospital for Diseases of the Chest has been published. He considers that the establishment of special hospitals for diseases of the chest has been justified. The utility of cod-liver oil in scrofulous disease is doubtless great, and the bacteriologists will perhaps give us further light on the relation between scrofula and tubercular phthisis. There is much, however, to learn of the relations between microbe and tubercle. We should not lose faith in old remedies. A case of pulmonary emphysema and bronchitis relieved by bleeding was cited. Sir James Bennett is convinced that in certain stages of acute disease, and in certain conditions of the patient, bleeding is a remedy of unquestionable value. The advantages gained by the aid of surgery in certain cases of chest disease have been demonstrated in the hospital, especially in the treatment of pleuritic effusions. Formerly paracentesis was only had recourse to in extreme cases. It is now available in numerous instances. If the bacillus theory is adhered to, a wide field is open for inquiry in what soils is the bacillus cultivable? There can be no doubt that phthisis must be classed as an hereditary disease. But it also appears that susceptibility may be acquired. Quoting Dr. Livingstone, Sir James Bennett mentioned that the black races in their own country rarely become phthisical, but in Europe they fall victims to phthisis. Jews especially are comparatively free from phthisis. But they are particular in their food, and never touch pork. Does the bacillus dislike this meat? The reduced mortality from phthisis, as shown in the Board of Health returns, seem to prove that this is due to improved sanitary conditions. If the microbe theory of phthisis were established, there seems no reason why protective inoculation should not prove as effective in one murrain as in another. We glean, however, that Sir James Bennett does not advocate this, also that he has some doubt as to the microbe theory of phthisis. Nevertheless, the facts mentioned in his address appear more favourable than otherwise to the truth of such theory.

